# Prospective cohort study of pregnancy complications and birth outcomes in women with asthma

**DOI:** 10.1007/s00404-018-4800-y

**Published:** 2018-05-24

**Authors:** Nasrin Fazel, Michael Kundi, Erika Jensen-Jarolim, Isabella Pali-Schöll, Asghar Kazemzadeh, Mojtaba Fattahi Abdizadeh, Habibollah Esmaily, Roya Akbarzadeh, Raheleh Ahmadi

**Affiliations:** 1 Center for Public Health, Medical University Vienna, Kinderspitalgasse 15, 1090 Vienna, Austria; 2 Department of Internal Medicine, University of Medical Sciences, Sabzevar, Iran; 3 Institute for Pathophysiology and Allergy Research, Medical University Vienna, Vienna, Austria; 4 The Interuniversity Messerli Research Institute, University of Veterinary Medicine Vienna, Medical University Vienna and University Vienna, Vienna, Austria; 5 Department of Microbiology, University of Medical Sciences, Sabzevar, Iran; 6 Department of Biostatistics and Epidemiology, Health Research Center, Mashhad University of Medical Sciences, Mashhad, Iran; 7 Paramedicine College, University of Medical Sciences, Sabzevar, Iran; 8 Department of Obstetrics and Gynecology, Mobini Hospital, University of Medical Sciences, Sabzevar, Iran

**Keywords:** Asthma, Pregnancy, Pregnancy complication, Birth outcome

## Abstract

**Background:**

Asthma is the most common potentially serious medical complication in pregnancy. The purpose of this study was to determine the association between maternal asthma and a spectrum of adverse neonatal and maternal outcomes.

**Methods:**

Events during pregnancy and birth outcome were evaluated in 34 asthmatic as well as 1569 non-asthmatic pregnant women who were enrolled in a prospective cohort study undertaken at the antenatal clinics of Mobini Hospital in Iran. The women were interviewed and classified according to clinical severity and asthma control as per GINA guidelines. Information on asthma symptoms was collected by a questionnaire as well as by spirometry and physical examination. All subjects were followed until delivery, and postpartum charts were reviewed to assess neonatal and maternal outcomes. Eosinophil cells counts were obtained and total IgE was measured by ELISA. Results were assessed by multivariate logistic regression adjusting for maternal age and parity, and for birth outcomes, for gestational diabetes, and hypertension/pre-eclampsia.

**Results:**

The well-known relationship between family history of asthma and asthma in pregnancy was again supported (*p* < 0.001). Women with asthma had more bleeding events 3 weeks or more before delivery (OR 3.30, 95% CI 1.41–7.26), more often placenta problems (OR 6.86, 95% CI 1.42–33.02), and gestational diabetes mellitus (OR 3.82, 95% CI 1.06–13.75). No significant differences between asthmatic and non-asthmatic mothers regarding duration of gestation, birthweight, low Apgar scores, or neonatal respiratory difficulties were found. Total IgE antibody levels and eosinophil counts did not differ by asthma control and severity.

**Conclusions:**

Asthma in pregnancy poses some risk for pregnancy complications and adverse perinatal outcomes. Managing asthma effectively throughout pregnancy could benefit women and their babies and help to reduce the health burden associated with asthma during pregnancy.

## Introduction

One of the most common respiratory diseases in young women is asthma [1]. The prevalence of asthma in women of childbearing age ranges from 0.4 to 12.4% [2–4], and it is estimated that between 4 and 8% of all pregnant women in the US suffer from this disease [5]. There are about 315 million people estimated to be suffering from asthma worldwide [6]. Moreover, the frequency of asthma in pregnancy (AP) is expected to increase in the coming decades [7]. Whereas many studies are focusing on how asthma affects pregnancy and vice versa, less is reported on effects of maternal asthma on the overall impact of asthma on pre- as well as neonatal outcomes. It is possible that the in utero environment is affected by the disease and hence could have an impact on the offspring. This is demonstrated by the fact that the development of asthma and atopy in children is more closely associated with maternal asthma or immunoglobulin (Ig)E levels than paternal asthma or IgE [8]. Studies investigating the long-term effects of asthma of mothers during pregnancy on the offspring have found an increase in wheezing incidence at 15 months of age and childhood respiratory diseases overall, while others found no developmental effects [9, 10]. However, several historical and prospective cohort and cross-sectional studies have been conducted on how pregnancy affects maternal asthma and vice versa [11–13].

Nevertheless, AP still remains an underappreciated condition in gynecological practice, even though reports on AP in this field are published since long [10, 14–16]. Hence, in light of the extent of AP worldwide and the scarcity of information about this disease, further studies are needed.

In a general obstetric population with asthma, the risk of almost all adverse pregnancy outcomes increases [17]. In some studies, uncontrolled asthma was associated with poor outcomes of pregnancy and adverse effects on the fetus, e.g., prematurity, low birthweight, and increased risk of congenital malformations (for a review, see [18, 19]). Impaired respiration during AP is a major concern in this regard. However, in earlier studies, the severity of maternal asthma had no direct effect on birth outcomes and pregnancy complications [20]. It was reported that careful management of asthma and its exacerbations during pregnancy may prevent most of the serious obstetric and neonatal disorders, thereby supporting the role of impaired oxygen supply as the source of adverse outcomes during AP [21–23]. Furthermore, the pros and cons of asthma medications must be weighed carefully against the risk of malformations in the child, especially when the mother has to be treated in the first trimester [24–26]. Too little information is available today on the risk of drugs during pregnancy for the child.

In the present study, we aimed to determine the association of AP with adverse events during pregnancy and pregnancy outcome in a large cohort of pregnant mothers in Iran.

## Materials and methods

### Study participants

Between August 2014 and April 2015, pregnant women were recruited from 18 obstetric clinics associated with Mobini Hospital in Sabzevar, Iran. They participated in a prospective study designed to examine the effects of asthma on pregnancy and pregnancy outcomes.

The prevalence of asthma in pregnant women varies between 2 and 12% in different countries. In an initial pilot study, in about 200 women, we estimated asthma prevalence to be 5%. This figure was used to estimate the sample size necessary to detect a twofold increased risk for complications with not too low incidences of 15% or more at a significance level of 5% and a power of 80%. The sample size was set to 1600 women, which is about 10% of all pregnant women in the city of Sabzevar in Iran in the period between August 2014 and April 2015.

Inclusion criteria were gestational age of week 24 or less. Excluded were those unable to understand the Persian language well enough to participate in an interview and consent to participate in the study, those with severe mental health problems, those with chronic obstructive pulmonary disease or other pulmonary diseases other than asthma, those with seasonal airway diseases, those having had sinus surgery within the previous 6 months, and those using systemic steroids in the past 4 weeks or longer for other diseases than asthma. The study was approved by the Sabzevar Medical University and the Human Investigations Committee.

### Study procedure

Since there were 18 obstetric clinics associated with Mobini Hospital, recruitment followed a cluster randomization procedure, with days of visit randomly allocated to the different clinics. At each visit day, all women present in the outpatient department that were before gestational week 24 were contacted and asked to participate if they fulfilled the inclusion criteria and had none of the exclusion criteria. Eligible pregnant mothers were then interviewed by trained staff using a standardized questionnaire.

Furthermore, a self-administered questionnaire to identify asthma or asthma-like symptoms was distributed to the women. The questionnaire was developed based on the International Union against Tuberculosis and Lung Disease (IUATLD) questionnaire [27]. It included 43 main questions in four sections: (a) asthma and asthma symptoms (12 questions, with sub-questions); (b) occupational exposures and medical history (17 questions, with sub-questions); (c) non-occupational exposures and asthma risk factors (9 questions); (d) demographics and obstetrics history (8 questions). It was translated into Farsi and assessed for accuracy, clarity, and comprehensibility in a preliminary study.

After the questionnaire was completed, an interview was conducted with all women already having been diagnosed with asthma, pregnant women who were currently taking one of the drugs commonly used to treat asthma, and pregnant women with symptoms of asthma revealed by the questionnaire (shortness of breath, coughing, and wheezing). The interview included a series of questions specifically related to asthma diagnosis, symptoms, and management covering the past 5 years. We asked about level of asthma control, whether asthma required unscheduled medical visits, hospitalization, or intubation. Furthermore, women were asked about interference of symptoms with daily activities or sleep and the Asthma Control Questionnaire was administered [28].

Asthma severity and asthma control were categorized as per GINA (Global Initiative for Asthma) guidelines [29].

All women with suspected asthma were transferred to a pulmonologist, specialized in asthma, and a spirometry was performed.

Follow-up interviews were managed by phone at 20 (if first interview was at least 4 weeks before), 28, and 36 weeks (± 5 days) of gestation and in the hospital following childbirth. In the follow-up interviews, detailed information about symptoms and medication use during pregnancy was collected.

Complications during pregnancy were extracted from the medical records and by interview after delivery. Pre-eclampsia was assigned if a previously normotensive patient had a systolic/diastolic blood pressure ≥ 140/90 mm Hg on at least two visits before week 20 and/or significant proteinuria and/or additional signs and symptoms that were indicative of pre-eclampsia. The diagnosis was established by vanishing of the hypertension after delivery. Premature rupture of membranes was defined as the leakage of amniotic fluid ≥ 24 h before the onset of labor and considered as complication if it occurred more than 3 weeks before delivery. Gestational diabetes was assigned if at least two of four oral glucose tolerance tests met or exceeded the upper limits of normal. We recorded every vaginal bleeding during pregnancy by interview: “Have you had any spotting or bleeding so far during this pregnancy?” and prompted to describe timing (gestational week) and heaviness (spotting, slight, about the same as usual period or heavier than usual period). Placenta problems included placenta praevia, placenta abruption, and placenta separation. Urinary tract infections during pregnancy comprised symptomatic and asymptomatic bacteriuria defined as more than 10^5^ colony-forming units per mL of urine.

Infants underwent physical examination by a neonatologist at least three times: at birth, at 1 day of age, and at hospital discharge (i.e., ≥ 5 days of age).

Complications of delivery were preterm delivery defined as birth before week 37 gestational age, non-elective cesarean section, low birthweight defined as birthweight less than 2500 g irrespective of gestational age, small for gestational age (SGA) defined as birthweight below the 10th percentile for the gestational age at delivery, Apgar score at 1 min equal or less than 7, respiratory difficulties defined as a child requiring at least short term respiration or transfer to a neonatal intensive care unit because of respiratory problems, and developmental anomalies or malformations (e.g., anencephaly, urethral stenosis, omphalocele, and spina bifida).

### Screening spirometry

Spirometry was performed using Spirolab ΙΙΙ S/N 000072 (MIR, Italy) applying the protocol and criteria of the American Thoracic Society. The patients were classified according to clinical severity, as per GINA guidelines, as suffering from intermittent asthma, mild persistent asthma, moderate persistent asthma, and severe persistent asthma [29].

### Blood sampling

Mothers’ blood samples were taken at the screening visit and neonates’ blood samples were drawn during hospital stay. All blood samples were stored in Mobini Hospital and sent to Mashhad Laboratory every week for measuring IgE and eosinophils.

### Statistical methods

Only about half the number of asthma cases was obtained than estimated from the pilot study. Therefore, the effects size that can be discriminated increased from an odds ratio of 2.0 to an odds ratio of 2.7. Statistical comparisons of groups were done by independent sample methods, including Student’s *t* test, Fisher’s exact test, Chi-square test, and Mann–Whitney *U* test, as appropriate. It turned out that groups of women with and without asthma differed in age and hence in parity and other age-related variables as well as by ethnicity, education, and area of living. In addition, women with asthma had more often a history of allergies and a family history of asthma. Except for the latter attributes, all other variables by which asthmatic and non-asthmatic women differed were considered as possible confounders. It turned out that among these potential confounders only age and parity had a relationship with outcomes and, therefore, the potential to confound the associations investigated. Therefore, all analyses were done adjusting for age and parity. In addition, several birth outcomes were adjusted for gestational diabetes and hypertension/pre-eclampsia. Outcomes were analyzed by generalized linear models (GLM) with binomial counts and a log link. Analyses were performed by SPSS 23.0 (IBM corp., USA).

## Results

### Description of cohort

Overall, 1609 women were enrolled prior to the 24th week of gestation (mean ± standard deviation 12 ± 3 weeks). Six women had asthma according to the questionnaire but did not consent to blood draw and spirometry. Results were obtained from 1603 pregnant women, of which 42 (2.6%) were suspected to have asthma. One of these patients was diagnosed with cardiovascular disease, six others were found to have respiratory disorders other than asthma, and one woman had an abortion in the ninth week of gestation. A total of 2.1% (34) were finally diagnosed with asthma, 10 of which were newly diagnosed. Among these 34 pregnant women with asthma, 13 (38.2%) were categorized as having intermittent asthma, 10 (29.4%) mild persistent asthma, 8 (23.5%) moderate persistent asthma, and 3 (8.8%) severe persistent asthma at the baseline examination. Demographic and obstetric data of the patients are shown in Table 1. Women with asthma were significantly older, and had previously had more pregnancies, including those aborted. Asthmatic women were significantly more often of Turk origin, lived in a village and were less educated. In addition, they had a much higher percentage of a history of asthma in the family (*p* < 0.001) and more often had a history of allergies (*p* < 0.001). From the 34 women with asthma, 5 took currently no medication, 8 were on combination therapy, and the majority was taking only one medication (Table 2).

**Table 1:** Sociodemographic and obstetric characteristics of asthmatic and non-asthmatic women (percentages or mean ± standard deviation)

Variable	Non-asthmatic women (*n* = 1569)	Asthmatic women (*n* = 34)	*p* value
Previous stillbirth(s)	1.6%	2.9%	0.430^a^
Previous ectopic pregnancy	0.4%	2.9%	0.158^a^
Previous hydatidiform mole(s)	0.3%	0.0%	1.000^a^
Previous abortion(s)	21.5%	38.2%	0.033^a^
Gravidities			
1	34.0%	14.7%	0.006^b^
2	34.2%	29.4%	
3	19.3%	26.5%	
> 3	12.4%	29.4%	
Parity			
First	40.7%	26.5%	0.017^b^
Second	37.4%	29.4%	
Third	17.3%	32.4%	
> Third	4.5%	11.8%	
Weight gain (kg)	12.1 ± 4.4	12.6 ± 5.7	0.866^c^
Gestational age (weeks)	38.9 ± 1.9	39.4 ± 1.2	0.339^c^
Age (years)	27.3 ± 5.9	31.0 ± 6.4	0.001^c^
Education			
Illiterate	1.6%	5.9%	0.029^b^
Elementary	19.7%	29.4%	
Pre-high school	21.4%	29.4%	
High school/college	57.3%	35.3%	
Employed	5.5%	8.8%	0.435^a^
Ethnicity			
Persian	84.3%	55.9%	< 0.001^b^
Turk	14.2%	44.1%	
Other	1.5%	0%	
Living in			
City	68.5%	47.1%	0.014^a^
Village	31.5%	52.9%	
Family history asthma	1.0%	26.5%	< 0.001^a^
Any allergy	12.7%	94.1%	< 0.001^a^

^a^Fisher’s exact probability test

^b^Chi-square test

^c^Mann–Whitney *U* test

**Table 2:** Anti-asthmatics taken by pregnant women with asthma (*n = *34)

Drug	*n*
Salbutamol	19
Fluticasone + salmeterol	4
Theophylline + salbutamol	2
Salbutamol + fluticasone	2
Benzyl benzoate	1
Beclomethasone	1

### Events during pregnancy

Some relevant events during pregnancy were significantly associated with asthma. Vaginal bleeding 3 weeks or more before delivery was noted in more than 23% of asthmatic women, while this occurred in only 8.5% of non-asthmatic women (*p* = 0.005). Placenta problems occurred in 5.9 vs. 0.8% in asthmatic vs non-asthmatic women, respectively (*p* = 0.016). In addition, gestational diabetes was more frequent (8.8 vs. 1.7%) in asthmatic women (*p* = 0.04) compared to non-asthmatic ones. Other conditions like pre-eclampsia, PROM, vomiting, or infections were not statistically different in asthmatic women compared to non-asthmatic controls (Table 3).

**Table 3:** Events during second and third trimesters of pregnancy in asthmatic and non-asthmatic women

Complication	Non-asthmatic women (*n* = 1569) %(adj%)	Asthmatic women (*n* = 34) %(adj%)	OR	95% CI	*p* value
Bleeding > 3 weeks before del.	8.5 (8.5)	23.5 (22.9)	3.20	1.41–7.26	0.005
UTI	9.9 (9.9)	11.8 (12.3)	1.28	0.44–3.71	0.647
Vomiting	20.7 (20.3)	17.6 (18.8)	0.91	0.37–2.23	0.834
Cerclage	0.9	0.0	–	–	0.705
HBP/PE	4.7 (3.8)	5.9 (3.2)	0.84	0.19-3.67	0.814
Placenta problem	0.8 (0.7)	5.9 (4.8)	6.86	1.42-33.02	0.016
PROM > 3 weeks before del.	4.3 (4.1)	2.9 (2.6)	0.63	0.08-4.68	0.648
GDM	1.7 (1.4)	8.8 (5.2)	3.82	1.06-13.75	0.040

Crude percentages and percentages adjusted by age and parity are shown. Odds ratios (OR), 95% confidence intervals (CI), and *p* values from logistic regression analysis

*UTI* urinary tract infection, *HBP/PE* high blood pressure/pre-eclampsia, *PROM* premature rupture of membranes, *GDM* gestational diabetes mellitus

### Birth outcome

Birthweight of children delivered by asthmatic women was non-significantly higher than those born by non-asthmatic women consistent with their slightly higher gestational age at birth. Asthmatic women had more often non-elective cesarean deliveries (*p* = 0.027). Preterm birth, low birthweight, and a low Apgar score were rather less frequent in women with asthma, although no significant differences were found. Newborns to asthmatic women were more frequently admitted to a neonatal care unit and had more respiratory problems, and there was a tendency (*p* = 0.098) for higher rates of anomalies (2.9 vs. 0.4%) in women with asthma compared to those without, but this was due to a single case among women with asthma (Table 4).

**Table 4:** Birth outcome and complications in asthmatic and non-asthmatic women

Complication	Non-asthmatic women (*n* = 1569) mean ± SE or %(adj%)	Asthmatic women (*n* = 34) mean ± SE or %(adj%)	OR	95% CI	*p* value
Birthweight (g)	3147 ± 12	3289 ± 80	–	–	0.081
Gender (males)	50.2 (50.2)	47.1 (47.2)	0.89	0.45–1.76	0.730
Preterm delivery	7.7 (7.7)	0.0 (0.0)	–	–	0.092
Non-elective cesarean	33.0 (34.2)	52.9 (46.9)	1.81	1.01–3.50	0.027
Low birthweight	5.9 (5.9)	2.9 (3.0)	0.49	0.07–3.65	0.488
Small for gestational age	2.7 (2.7)	2.9 (2.9)	1.08	0.14–8.15	0.942
Apgar 1 min ≤ 7	13.4 (13.3)	5.9 (5.6)	0.39	0.09–1.63	0.194
Respiratory difficulties	3.1 (3.0)	5.9 (5.5)	1.88	0.43–8.14	0.401
Admission of infant	7.0 (6.7)	11.8 (11.1)	1.73	0.59–5.06	0.315
Anomaly^a^	0.4 (0.4)	2.9 (2.7)	6.22	0.71–54.22	0.098

Except for birthweight, crude percentages and percentages adjusted for age and parity (analysis of birthweight, preterm delivery, non-elective caesarean, low birthweight, and small for gestational age were additionally adjusted for gestational diabetes, hypertension, and pre-eclampsia) are shown. Odds ratios (OR), 95% confidence intervals (CI), and *p* values from logistic regression analysis

*SE* standard error

^a^Anencephaly, urethral stenosis, extra finger, omphalocele, kidney cyst, and spina bifida

### Asthma severity and asthma control

We dichotomized asthma control into those with controlled asthma (*n* = 13) and those with partly or poorly controlled asthma (*n* = 21). Asthma severity was categorized into intermittent or mild persistent asthma (*n* = 23) and those with moderate or severe persistent asthma (*n* = 11). Those with controlled asthma as well as those with less severe asthma were less frequent on combination therapy. No difference with respect to asthma control and severity was found concerning eosinophils counts and IgE levels (Table 5). No statistically significant differences in frequency of events during pregnancy and delivery were seen between women with controlled asthma compared to partly or poorly controlled conditions (Table 6). A significantly higher (*p* = 0.007) rate of urinary tract infection was found in women with more severe asthma. More women in the group with moderate or severe persistent asthma experienced emesis (*p* = 0.07) and also a higher frequency (*p* = 0.098) of Apgar ≤ 7 occurred in the 1st min after delivery in these women (Table 6).

**Table 5:** Asthma control and severity related to number of anti-asthmatics taken, lung function (FEV1), eosinophil counts, and IgE levels

	Controlled (*n* = 13)	Partly/poorly controlled (*n* = 21)	*p* value^a^	IMPA (*n* = 23)	MSPA (*n* = 11)	*p* value^a^
Anti-asthmatics						
1 medication	11 (85%)	15 (71%)	0.444	19 (82.6%)	7 (63.6%)	0.388
> 1 medication	2 (15%)	6 (29%)		4 (17.4%)	4 (36.4%)	
FEV1%						
80%	11 (85%)	16 (76%)	0.682	19 (82.6%)	8 (72.7%)	0.656
< 80%	2 (15%)	5 (24%)		4 (17.4%)	3 (27.3%)	
Eosinophils						
5%	12 (92%)	21 (100%)	0.382	22 (95.7%)	11 (100.0%)	1.000
> 5%	1 (8%)	0		1 (4.3%)	0 (0.0%)	
IgE						
120 U/mL	9 (69%)	15 (71%)	1.000	16 (69.6%)	8 (72.7%)	1.000
> 120 U/mL	4 (31%)	6 (29%)		7 (30.4%)	3 (27.3%)	

*IMPA* intermittent/mild persistent asthma, *MSPA* moderate/severe persistent asthma

^a^Fisher’s exact probability test

**Table 6:** Events during pregnancy and birth outcomes by asthma control and asthma severity

Complication	Controlled (*n* = 13) (%)	Partly/poorly controlled (*n* = 21) (%)	*p* value	IMPA (*n* = 23) (%)	MSPA (*n* = 11) (%)	*p* value
Bleeding	15.4	28.6	0.444	17.4	36.4	0.388
UTI	0.0	19.0	0.144	0.0	36.4	0.007
Vomiting	7.7	23.8	0.370	8.7	36.4	0.070
HBP/PE	7.7	4.8	1.000	4.3	9.1	1.000
Placenta problem	0.0	9.5	0.513	4.3	9.1	1.000
PROM	0.0	4.8	1.000	4.3	0.0	1.000
GDM	15.4	4.8	0.544	13.0	0.0	0.535
Low birthweight	0.0	4.8	1.000	0.0	9.1	0.324
Small for gest. age	0.0	4.8	1.000	0.0	9.1	0.324
Apgar 1 min ≤ 7	0.0	9.5	0.513	0.0	18.2	0.098
Respiratory diff.	15.4	0.0	0.139	8.7	0.0	1.000
Anomaly	0.0	4.8	1.000	0.0	9.1	0.324

*p* values from Fisher’s exact test

*IMPA* intermittent/mild persistent asthma, *MSPA* moderate/severe persistent asthma, *UTI* urinary tract infection, *HBP/PE* high blood pressure/pre-eclampsia, *PROM* premature rupture of membranes, *GDM* gestational diabetes mellitus

## Discussion

In the present study, we looked into the possible connection between asthma and pregnancy problems as well as birth outcome. Women with asthma had more bleeding events 3 weeks or more before delivery (OR 3.30, 95% CI 1.41–7.26), more often placenta problems (OR 6.86, 95% CI 1.42–33.02), and gestational diabetes (OR 3.82, 95% CI 1.06–13.75). This is in line with a large US American retrospective cohort study that, in addition, revealed an association with pre-eclampsia too [30]. We did not find a higher prevalence of pre-eclampsia in the asthmatic group than in non-asthmatic women (3.2 vs 3.8%), which might be due to the lower fraction of moderate and severe persistent asthma (32%) in our study. Duration of gestation, birthweight, low Apgar scores, and neonatal respiratory difficulties were not significantly different between the asthmatic and control subjects. In accordance with a previous study [31], we report a tendency towards higher incidence of infant malformation among live births by asthmatic women. Another study reports a significantly increased incidence of congenital malformations in asthmatic women with exacerbations during the first trimester [32].

There was also an increased incidence of caesarean delivery in asthmatic women. One possible reason for this is that pregnant asthmatics are considered high-risk patients, and so are more likely to undergo surgery, as compared to healthy pregnant women [33]. In this study [33], caesarean was performed in 52.9 and 34.2% of asthmatics and non-asthmatics, respectively, in close agreement with our results of 52.9 and 33.0%. Our results are also comparable to those of Dombrowski et al. [34], where the highest rate of caesarean sections occurred in cases of intermittent and mild persistent asthmatics, at the rates of 53.8 and 70.0%, respectively.

All women with asthma had a baby with a gestational age in the category 37–42 weeks. These results are comparable to the study of Firoozi et al. [35]. Especially, with severe and poorly controlled asthma, a relationship between fetal growth retardation and asthma has been reported that could be due to the compromised oxygen saturation of hemoglobin in asthmatics, which should be above 90% during pregnancy to guarantee sufficient fetal oxygenation [36, 37]. A possible reason that we could not find indications of fetal growth retardation may be found in the fact that the majority of women were on asthma medication and although not perfectly controlled the worst consequences of impaired breathing and of wheezing episodes might have been avoided [24–26].

The health of the newborn infants of asthmatics was generally good. We found no significant difference between the neonates of asthmatic and non-asthmatic women regarding any of the birth outcome categories. An almost doubled incidence of respiratory problems in newborns from asthmatic women was statistically not significant.

We also found no difference in frequency of events during pregnancy and in pregnancy outcome between categories of asthma severity and asthma control. While there is evidence from earlier studies that poorly controlled and severe persistent asthma is associated with intrauterine growth retardation and, consequently, with lower birthweight and other adverse birth outcomes [38], it has also been shown that treatment of asthma attacks reduces risks for adverse birth outcomes [39, 40]. Although this was not an interventional study, for ethical reasons, all women with suspected asthma were transferred to an asthma specialist who checked and adapted, if necessary, asthma medication.

The prevalence of asthma in pregnancy in our study was 2.1%. Most reported AP prevalences are in the range of 2–12%. In a 2007 study, Karimi et al. [31] identified 5.6% of pregnant women in Iran as being asthmatic. The prevalence found in our study is at the lower limit of the reported range. In general, asthma prevalence depends on geographical area, local environmental and climatic conditions, lifestyle factors, vegetation, and air pollution. Demissie reported that asthma affected between 0.4 and 1.3% of pregnant women in New Jersey [41], whereas the prevalence of AP in Australia was calculated to be 12% [33]. It is also possible that variations in asthma prevalence are in part due to epigenetic factors and environmental exposures during the early childhood [16].

One limitation of our study was that despite the large sample of pregnant women, in only few of them, asthma could be diagnosed. Although the prevalence of 2.1% falls within the range reported in the literature, it is rather at the low end and about half the number which we expected. As a consequence, subdivision into asthma severity categories or asthma control resulted in too low numbers of women to be meaningfully analyzed. However, the strength of this study is that a cohort of women from a region including densely populated areas and rural districts was followed during pregnancy and the first week after delivery and hence is among the very few prospective studies of asthma in pregnancy.

To conclude, maternal asthma, although of low prevalence, appeared to be a major risk factor for some complications of pregnancy, in particular, vaginal bleeding, placenta problems, and gestational diabetes. It seems that these complications do not lead to problems of delivery or adverse birth outcomes with the possible exception of respiratory problems in the offspring and congenital malformations that occurred in one child of a woman with asthma when 1 in about 250 births would be expected.
